# Effect of sterilization on the cutting efficiency of two different rotary NiTi instruments (An In- vitro Study)

**DOI:** 10.1186/s12903-025-06534-w

**Published:** 2025-07-16

**Authors:** Merna Mamdouh Botros, Kariem Mostafa ElBatouty, Tariq Yehia Abdelrahman

**Affiliations:** Endodontics, Faculty of Dentistry, Ain Shams University, Cairo, Egypt

**Keywords:** NiTi, Rotary files, Sterilization, Cutting efficiency

## Abstract

**Background:**

Dental rotary instruments can be applied under multiple canal conditions. This study assessed the performance of two batches of NiTi dental rotary files—Mani Jizai (Jz) and Dentsply ProTaper Next (PTN)—by evaluating their cutting efficiency.

**Methods:**

A total of 80 files (40 from each type), with 8x digital microscope magnification used for defect inspection, were tested half of which before and the other half after sterilization. The sample size was tested in extracted upper molars’ distobuccal (DB) roots to assess cutting efficiency by volumetric changes showing removed dentin volume.

**Results:**

The Results indicated that the sterilized PTN files performed better at 5 mm, but no significant differences were noted at 10 mm between all file groups. At 15 mm, the performance of sterilized PTN files was inferior to that of non-sterilized Jz files.

**Conclusion:**

Sterilization and heat treatment affect file cutting efficiency by volumetric changes, especially at the coronal and apical one-thirds.

## Background

Effective disinfection relies on properly shaping the root canals to optimize irrigants and medicaments. However, the complexity of root canal anatomy and instrument limitations make this task difficult [1]. Since their introduction, nickel-titanium (Ni-Ti) alloys have significantly advanced the field of endodontics [2]. Research indicates that engine-driven Ni-Ti endodontic instruments achieve more precise root canal preparation, causing less canal transportation and fewer preparation errors than stainless steel hand instruments [3]. 

In clinical practice, NiTi files are often reused and subjected to autoclave sterilization. While single-use is ideal from a safety standpoint, reusing sterilized NiTi files is still common due to cost, durability perceptions, and effective sterilization methods. However, this practice requires careful inspection and risk assessment [4]. Studies have shown that the additional heat exposure during sterilization can influence their mechanical properties and flexibility [5]. 

Cutting efficiency in endodontic instruments is crucial for reducing working time, simplifying systems, and preventing torsional overload. Instruments with higher cutting efficiency require less torque and apical force, improving root canal preparation. Research on new NiTi (Nickel-Titanium) systems has focused on how flute design, kinematics, and thermal treatments affect performance. Post-machining treatments and autoclaving can influence NiTi alloy properties [6]. 

Instruments such as PTN, made from M-Wire, have become popular because of their improved mechanical properties [6]. Similarly, the Jz system, made from another heat-treated NiTi alloy, offers better fatigue resistance and features such as an off-center cross-section that reduces screw-in forces and improves debris removal [7]. 

Therefore, evaluating how repeated autoclaving cycles impact cutting efficiency of martensite and controlled memory (M- and CM- Wire) files, PTN and Jz, respectively, is valuable.

The null hypothesis tested was that there is no difference in cutting efficiency of new and sterilized PTN and JZ instruments.

## Methods

### Sample size calculation

Power analysis was designed to have adequate power to apply a statistical test of the null hypothesis that there is no difference would be found between different tested groups regarding cutting efficiency by volume. By adopting an alpha (α) level of (0.05), a beta (β) of (0.2) (i.e. power = 80%), and an effect size (f) of (0.595) calculated based on the results of a previous study; the sample size (n) was found to be a total of (80) samples (i.e. 40 samples per group, 20 samples per subgroup). Sample size calculation was performed using G*Power version 3.1.9.7. (Faul, Franz, et al. “G* Power 3: A flexible statistical power analysis program for the social, behavioral, and biomedical sciences.” Behavior research methods 39.2 (2007): 175–191) [8, 9]. 

### Ethical aspects and samples

This study was exempt from ethical review by the Ain Shams University Research Ethics Committee (FDASU-REC), [ethics committee approval: FDASU-Rec EM112308], as it was an in vitro study that used natural sound teeth collected from anonymous patients who consented to the use of their extracted teeth for research purposes, and it’s a routine process as they are treated in an educational hospital.

### Sample size classification (Fig. 1)

**Fig. 1:**
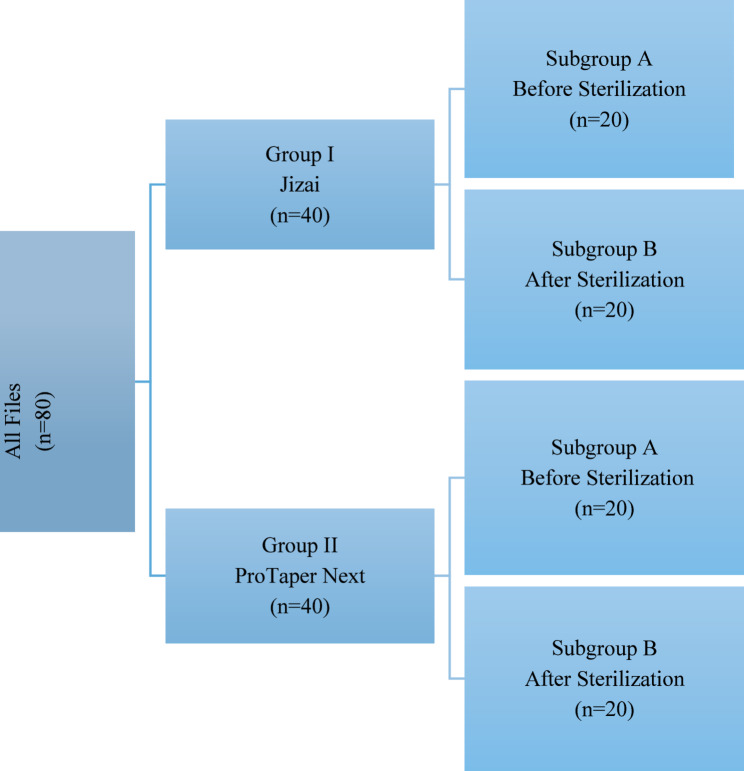
Samples classification

### Sample preparation

#### Sterilization

Half of the sample size, Forty rotary files (20 Jz files and 20 PTN files) were sterilized via the Ritter CleanTec AB80 (Ritter Concept GmbH, Biberach, Germany) Universal Program (134 degrees Celsius, 2 bars/29 psi, 30 min operating time and 20 min drying time) [10]. Sterilization was done prior to the use of files. Each file was used once.

#### Cutting efficiency

**Fig. 2:**
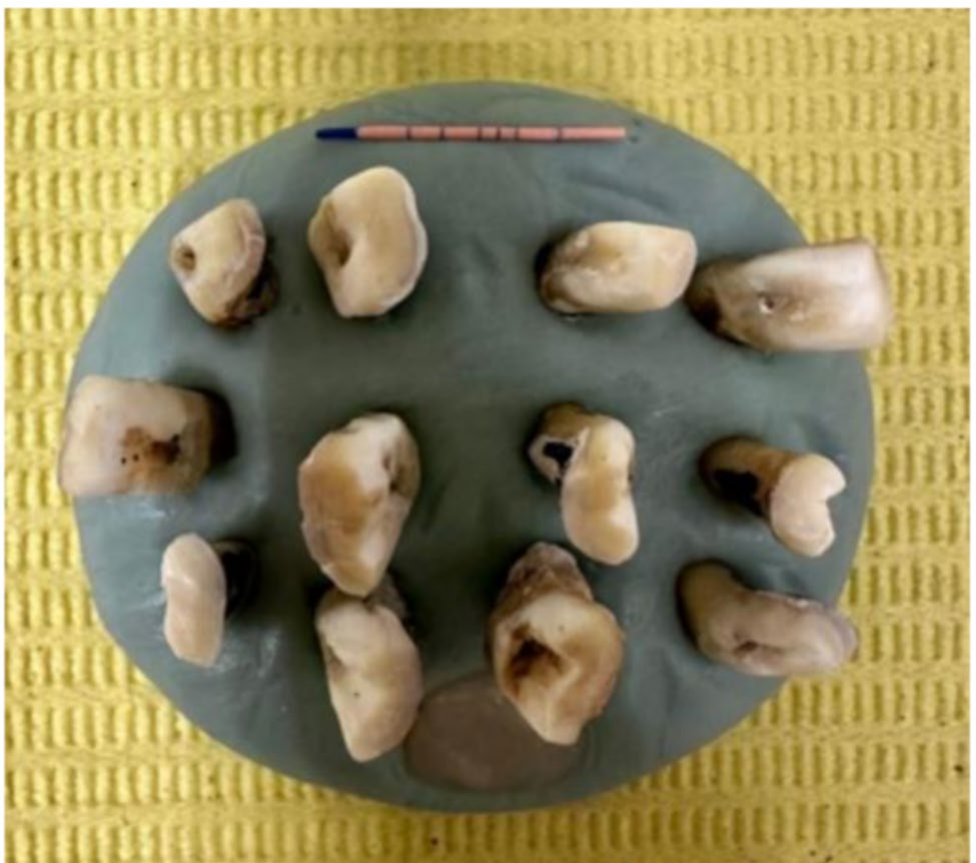
Set rubber base with sectioned roots aligned

The study involved cleaning recently extracted teeth and storing them in saline for no more than 30 days until use to avoid dehydration [11]. Standard access cavities were created in each pulp chamber, and working length (WL) was determined by introducing a size #10 K-file into the canals. The distobuccal (DB) roots were then separated and decoronated from extracted sound maxillary molars, with the length standardized to 16 mm [12, 13]. The teeth were embedded in rubber blocks to standardize their alignment for 3D tomography imaging before and after instrumentation [14]. (Fig. 2)

The canals were prepared using a crown-down technique and irrigated with sodium hypochlorite. Then apical patency was re-checked using #10 K-file [15]. Forty canals were prepared with JZ rotary files, while the other forty were prepared using PTN rotary files, with half of each group using non-sterilized files and the other half using sterilized ones.

The remaining dentin thickness was measured before and after preparation using tomographic scans. The entire procedure was conducted by one operator, while a separate examiner, blinded to the experimental groups, assessed the root canal curvatures and dentin thickness. Each set of instruments was used only once and then discarded.

The study used Cone-Beam Computed Tomography (CBCT) to measure canal curvature and evaluate root canal preparation. Pre- and post-operative CBCT scans of the teeth were performed with a Gendex Radiographic System (GXDP-800^®^), and the images were reconstructed with OnDemand 3D software (OnDemand3D™ app, Cybermed Inc., Seoul, South Korea (153–769). The rubber base block was positioned on the CBCT machine for standardization, with patient positioning lights adjusted to align the region of interest (ROI) in the field of view [15, 16]. 

Canal curvature was measured using Schneider’s technique [17] on the CBCT images, where the angle between two lines drawn along the canal was assessed. The homogeneity of the subgroups regarding canal curvature was statistically insignificant. Radiographic examination confirmed that the DB root had a single, patent, separate canal with a curvature of less than 25°, as assessed using the Schneider method (1971) [13, 17]. 

**Fig. 3:**
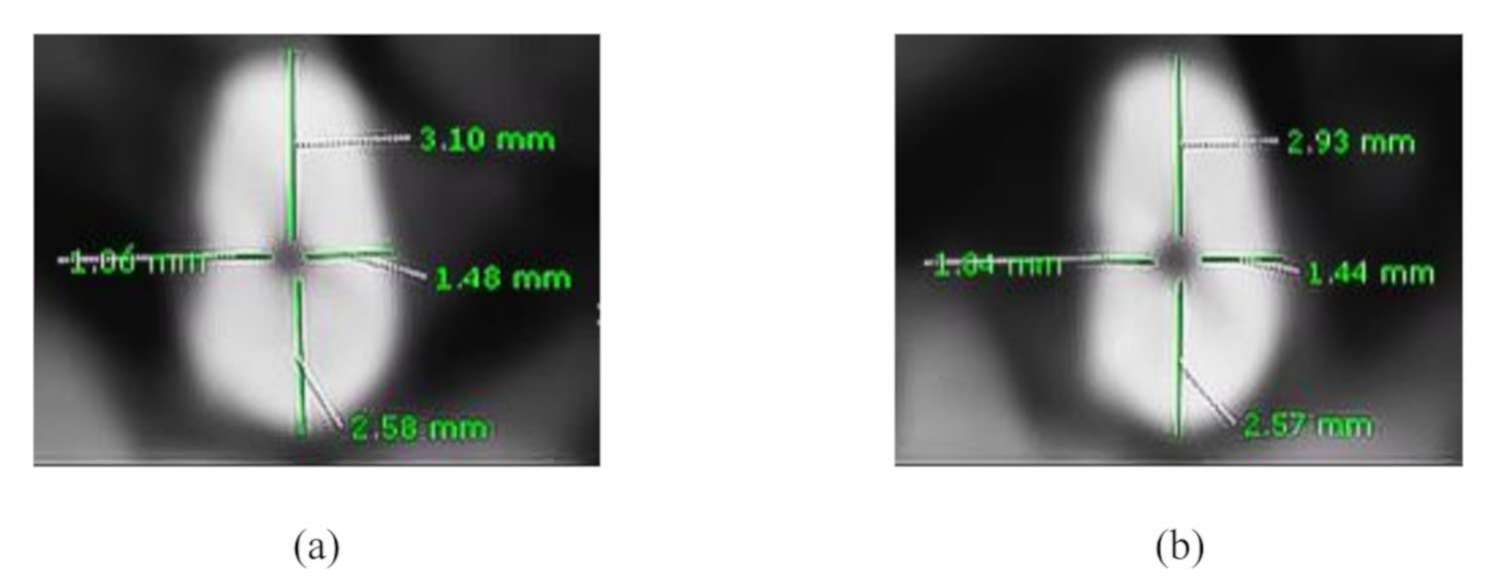
Method of measuring the pre- and postoperative dentin thickness where (**a**) and (**b**) represent the pre and postoperative dentin thickness measures respectively

For evaluating cutting efficiency, dentin thickness was measured at three levels (apical, middle, and coronal) of the DB canal before and after instrumentation. Dentin thickness was measured in mesial, distal, buccal, and lingual directions. The amount of dentin removed was calculated by subtracting post-instrumentation measurements from pre-instrumentation values (Y1-Y’1, Y2-Y’2, X1-X’1, X2-X’2) for each direction at each level and then an average value was calculated for each canal level and for each file. This assessment allowed the quantification of dentin removal at different sections of the canal. (Fig. 3) [16].

### Statistical analysis

Categorical data will be represented as frequency (n) and percentage (%) and will be analyzed using chi square test. Numerical data will be explored for normality by checking the data distribution, calculating the mean and median values and using Shapiro-Wilk test. If the data was found to be normally distributed, it would be presented as mean and standard deviation values and three-way ANOVA followed by Tukey’s post hoc test will be used for the analysis. If the assumption of normality was found to be violated, the data will be presented as median and range values and will be analyzed using Kruskal-Wallis test followed by Dunn’s post hoc test with Bonferroni correction. The significance level will be set at *p* ≤ 0.05 for all tests. Statistical analysis will be performed with IBM (IBM Corporation, NY, USA) SPSS^®^ (SPSS, Inc., an IBM Company) Statistics Version 26 for Windows [18, 19]. 

## Results

### Effect of file type

At 5 mm, the highest mean was seen in group non-sterilized PTN (0.18 ± 0.07) and the lowest mean was seen in non-sterilized PTN (0.064 ± 0.02). The highest volume of dentin removal was seen in group sterilized PTN, which was highly significantly different than non-sterilized Jz. There was insignificant difference among the other groups.

At 10 mm, the highest mean was seen in group sterilized PTN (0.133 ± 0.07) and the lowest mean was seen in group non-sterilized Jz (0.072 ± 0.03). There was an insignificant difference between the four groups.

At 15 mm, the highest mean was seen in group non-sterilized Jz (0.1 ± 0.04) and the lowest mean was seen in group sterilized PTN (0.058 ± 0.03). There was a significant difference between sterilized PTN and non-sterilized Jz. There was insignificant difference among the other groups.

### Effect of canal level

In non-sterilized Jz, the highest mean was seen at the apical root level (0.1 ± 0.04) and the lowest mean was seen at the middle root level (0.072 ± 0.03), showing no significant difference between both.

In sterilized Jz, the highest mean was seen at the middle root level (0.124 ± 0.09) and the lowest mean was seen at the apical root level (0.068 ± 0.03), showing no significant difference between both.

In non-sterilized PTN, the highest mean was seen at the middle root level (0.077 ± 0.03) and the lowest mean was seen at the coronal root level (0.064 ± 0.02), showing no significant difference between both.

In sterilized PTN, the highest mean was seen at the coronal root level (0.18 ± 0.07) and the lowest mean was seen at the apical root level (0.058 ± 0.03). There was a significant difference between both.

**Fig. 4:**
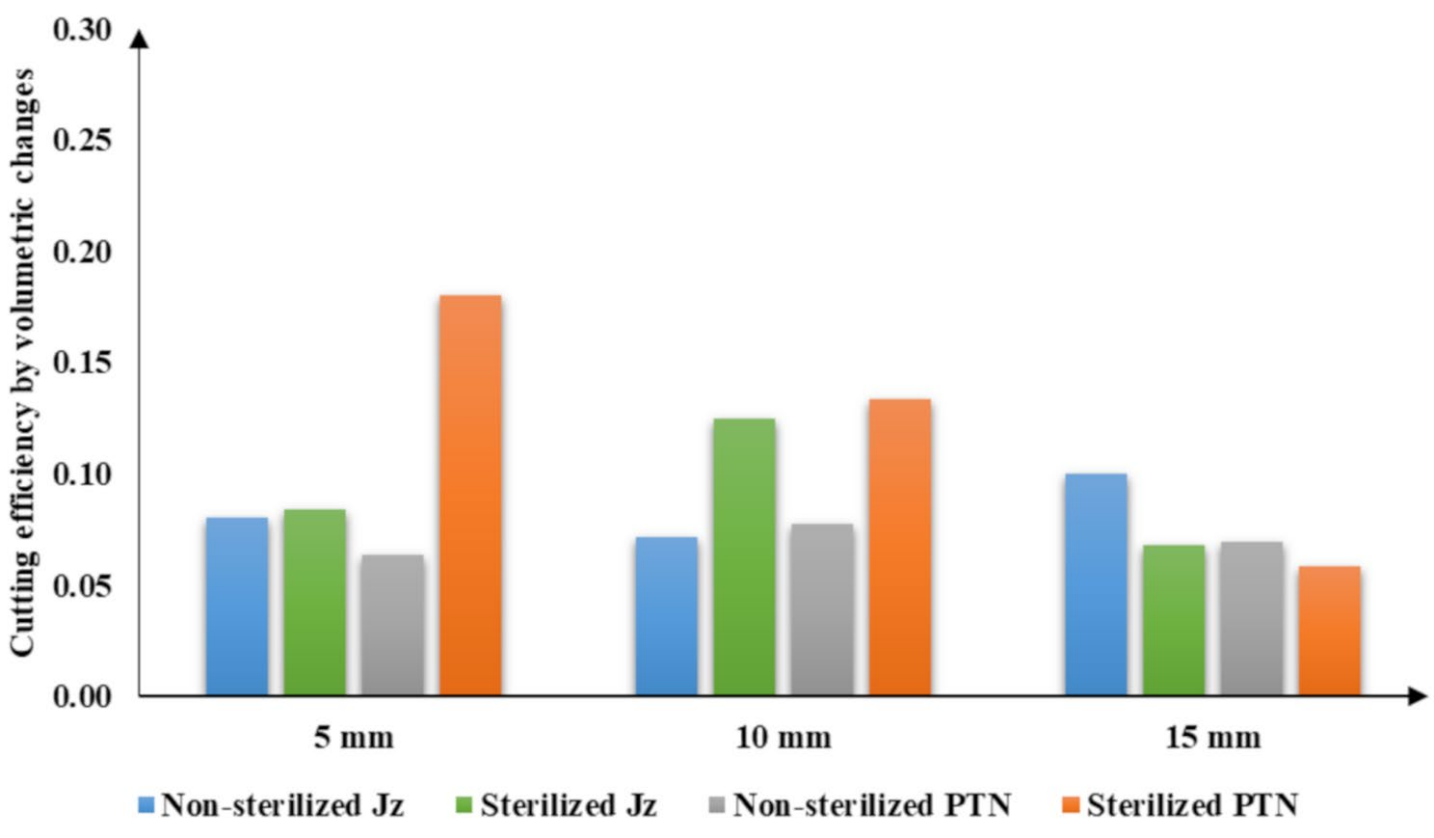
Bar chart showing volumetric changes to assess cutting efficiency of the study groups (mm^3^) at 5-, 10- and 15-mm root levels

**Fig. 5:**
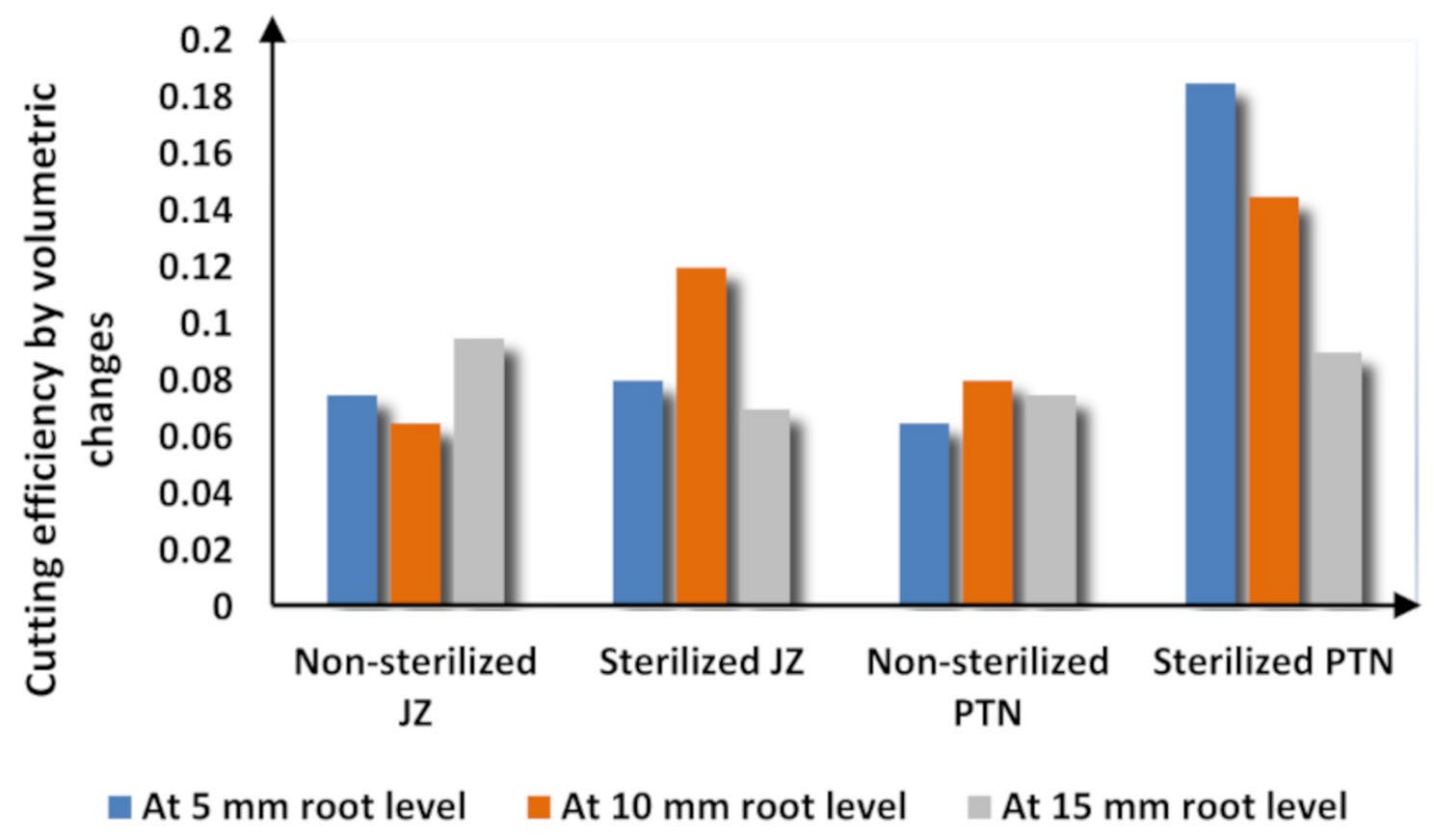
Bar chart showing difference in volumetric changes to assess cutting efficiency of the study groups (mm^3^) at 5,10-, and 15-mm root levels

Overall, the highest mean was in group sterilized PTN at the coronal level (0.18 ± 0.07) and the lowest mean was in group sterilized PTN at the apical level (0.058 ± 0.03), showing a significant difference between both. (Table 1) (Fig. 4) (Fig. 5).

**Table 1:** Mean ± SD of volumetric changes of the study groups (mm^3^)

		Non-sterilized JZ	Sterilized JZ	Non-sterilized PTN	Sterilized PTN	*P* value
5 mm (coronal)		0.080 ± 0.04^Aa^	0.084 ± 0.03^Aa^	0.064 ± 0.02^Aa^	0.18 ± 0.07^Bb^	< 0.001*
10 mm (middle)		0.072 ± 0.03^Aa^	0.124 ± 0.09^Aa^	0.077 ± 0.03^Aa^	0.133 ± 0.07^Aa^	> 0.05
15 mm (apical)		0.1 ± 0.04^Aa^	0.068 ± 0.03^Aab^	0.069 ± 0.01^Aab^	0.058 ± 0.03^Bb^	< 0.05*
*P* value		> 0.05	> 0.05	> 0.05	< 0.05*	

Significant difference at *P* ≤ 0.05

Similar superscript uppercase letter denotes insignificant difference in each column

Similar superscript lowercase letter denotes insignificant difference in each row

## Discussion

Biomechanical root canal preparation has evolved from manual techniques to rotary NiTi instruments, improving consistency, predictability, and efficiency, particularly for curved canals. Heat treatment enhances these instruments’ flexibility and fracture resistance. Cutting efficiency is key, helping with effective dentin removal and reducing torsional fractures. NiTi files function laterally (brushing) and axially to reach the working length [20, 21]. 

This study explored how autoclave sterilization affects the cutting efficiency of heat-treated NiTi files (Jz and PTN). Heat treatment (M-wire for PTN and CM-wire for Jz) is known to improve the mechanical properties of files, although the specific methods used by manufacturers are not disclosed [22]. Previous studies on autoclaving effects have shown mixed results, with some reporting no impact and others reporting a decrease in fatigue cycles after sterilization [5]. 

Cutting efficiency is measured using weight loss, debris generation, penetration depth, and time to reach the working length. In this study, cutting efficiency was evaluated using Cone Beam Computed Tomography (CBCT), which offers detailed 3D analysis of canal morphology, dentin thickness, and curvature, proving a reliable method for root canal evaluation [23]. 

Dentin removal was measured at coronal, middle, and apical root levels, with measurements in four directions to assess dentin cutting ability. CBCT imaging was used to assess dentin thickness and canal curvature, offering high precision with minimal specimen degradation. The study’s use of standardized root lengths and curvature, along with computer-controlled motor settings, ensured reliable and reproducible results. Irrigation with sodium hypochlorite and EDTA tri-sodium solution helped prevent smear layer compaction and maintain working length [24, 16, 25, 26, 27, 28, 29, 30]. 

Natural teeth were preferred over plastic blocks due to the more accurate replication of clinical conditions, as plastic lacks the texture and properties of dentin. The study found that NiTi files show less deformation in natural teeth than in resin blocks and using plastic can affect file shape and increase fracture risk. To account for variations in natural tooth morphology, experimental groups were balanced by root canal length and curvature angle, ensuring no significant differences between subgroups [13, 29]. 

Sterilized PTN rotary file showed the greatest cutting efficiency with significant difference among the other groups at 5 mm (coronal) root level. There was an insignificant difference between the other file groups at this level. The aggressiveness in cutting of PTN file may be attributed to its progressive tapering design which makes its cutting at the coronal level to be higher than the middle and apical levels, and this agreed with many studies as those by Peet et al. [31] and Pier et al. [32] Coronal pre-flaring of root canals offers multiple benefits that enhance the cleaning and shaping process. One key clinical advantage is that it facilitates the insertion of both manual and rotary instruments and the irrigant into the apical portion of the canal for proper disinfection [33, 34]. Studies have shown that pre-flaring generally leads to more accurate working length measurements in most cases [35]. However, excessive coronal flaring could also have a detrimental effect on the peri-cervical dentin resulting in root weakening [36]. 

Ideally, a minimum of 1 mm of dentin should remain intact on all sides of the root throughout its entire length following root canal preparation. Therefore, the instrumentation process must allow for canal enlargement without compromising the structural integrity of the tooth. Preserving the amount and volume of dentin—particularly in the peri-cervical region—is crucial for maintaining the tooth’s resistance to fracture [36, 37]. 

There was an insignificant difference between all four file groups at 10 mm (middle) root level. This may be related to the asymmetrical cross-sectional design and the 6% taper of the files that increases the core metal of the file and so increases the aggressiveness of the file especially at the middle part [38, 39]. 

Sterilized PTN rotary file showed the lowest cutting efficiency with significant difference in comparison with new Jz rotary file at 15 mm (apical) root level. There was an insignificant difference between the other file groups at this level. The reduced cutting ability of PTN at the apical level could be attributed to the reduced taper apical than coronal as agreed with studies by Jason et al. [40] and Haider et al. [41] Reduced apical preparation can compromise cleaning effectiveness, prompting clinicians to adopt strategies that enhance antimicrobial treatment outcomes. These strategies may include improving irrigant efficacy, optimizing physical activation methods, and using intracanal medicaments to compensate for limited instrumentation and achieve optimal root canal disinfection [42]. The reduced apical cutting effectiveness also results in reduced debris extrusion and thus less postoperative pain [43]. 

The study had few limitations, such as varying factors such as operator skill and anatomical differences, as well as it was found that no file system fully prepared all root canal walls, emphasizing the need for clinicians to enhance antimicrobial treatment effectiveness. Strategies such as improving irrigant efficacy, optimizing activation techniques, and using intracanal medicaments are crucial to compensate for incomplete file preparation. The study’s in vitro design and the lower accuracy of CBCT compared to micro-CT were limitations, though CBCT was still useful for assessing unprepared areas. Future research should consider using micro-CT for more precise evaluations [41, 44]. 

Due to the challenges of in-vivo studies, extracted teeth were used to simulate clinical conditions; however, acrylic models lack features like pulp tissue and natural apical constriction, and the heat from rotary instruments may distort them, affecting results [45]. Further assessment of any interaction between sterilization and exposure to irrigants (as NaOCl exposure) on the measured cutting efficiency could also be a suggestion for future research; such interactions might synergistically or antagonistically affect cutting efficiency and other file properties [5, 46, 47, 48]. Single use file systems without the need for sterilization could also be a future study direction to eliminate the cumulative effect of multiple sterilization cycles on dentin removal capacity. Peraça et al. [4] and Kowalczuck et al. [49] found that heat achieved during autoclaving may diminish the cutting capacity of instruments.

The study did not evaluate the effect of sterilization on other critical mechanical properties essential for clinical success, such as cyclic fatigue resistance or torsional strength, which are paramount for preventing instrument fracture during clinical use, especially in narrow and curved canals [5, 10]. Furthermore, other aspects of shaping ability (e.g., canal transportation, centering ratio) were not evaluated.

## Conclusions

The following conclusions can be drawn from the results of this study:

1. Cutting efficiency at 5 mm root level was higher in sterilized PTN files than other groups and was insignificantly different among other groups.
2. Cutting efficiency at 10 mm was insignificantly different among the four groups.
3. Cutting efficiency at 15 mm root level was significantly lower in sterilized PTN files than non-sterilized Jz files and was insignificantly different among other groups.

### Glossary

- Rotary File: Endodontic instrument used to clean and shape the root canal during root canal treatment (RCT). Unlike manual files, which require hand motion to operate, rotary files are powered by an endodontic motor that rotates the instrument at high speeds.
- Sterilization: refers to the process of eliminating all forms of microbial life, including bacteria, viruses, fungi, and bacterial spores, from the files used in root canal treatment. This ensures that the files are entirely free of contamination before being used in the root canal system, where sterile conditions are critical to prevent introducing infections into the tooth.
- Cutting Efficiency: refers to the ability of an endodontic instrument (such as rotary or hand files) to effectively remove dentin and pulp tissue from the root canal system with minimal effort, time, and risk of file breakage.
- CBCT: Cone Beam Computed Tomography is a specialized type of X-ray imaging that provides three-dimensional (3D) images of the dental and skeletal structures.

## Data Availability

All the data analyzed during this study are included in this published article in the form of tables and figures.

## Abbreviations

NiTi: Nickel Titanium
Jz: Jizai
PTN: ProTaper Next
RCT: root canal treatment
HEDM: Hyflex electrical discharge machining
AFBS: AF Blue S
DB: Distobuccal
CBCT: Cone Beam Computed Tomography
GXDP: Gendex Radiographic System
ROI: Region of Interest
WL: Working Length
